# Cancer immunotherapy using PolyPurine Reverse Hoogsteen hairpins targeting the PD-1/PD-L1 pathway in human tumor cells

**DOI:** 10.1371/journal.pone.0206818

**Published:** 2018-11-06

**Authors:** Miriam Marlene Medina Enríquez, Alex J. Félix, Carlos J. Ciudad, Véronique Noé

**Affiliations:** Department of Biochemistry and Physiology, School of Pharmacy, and Institute of Nanoscience and Nanotechnology, University of Barcelona, Barcelona, Spain; University of South Alabama Mitchell Cancer Institute, UNITED STATES

## Abstract

Immunotherapy approaches stand out as innovative strategies to eradicate tumor cells. Among them, PD-1/PD-L1 immunotherapy is considered one of the most successful advances in the history of cancer immunotherapy. We used our technology of Polypurine reverse Hoogsteen hairpins (PPRHs) for silencing both genes with the aim to provoke the elimination of tumor cells by macrophages in co-culture experiments. Incubation of PPRHs against *PD-1* and *PD-L1* decreased the levels of mRNA and protein in THP-1 monocytes and PC3 prostate cancer cells, respectively. Viability of THP-1 cells and macrophages obtained by PMA-differentiation of THP-1 cells was not affected upon incubation with the different PPRHs. On the other hand, PC3 cell survival was partially decreased by PPRHs against *PD-L1*. The greatest effect in decreasing cell viability was obtained in macrophages/PC3 co-culture experiments by combining PPRHs against *PD-1* and *PD-L1*. This effect was also observed in other cancer cell lines: HeLa, SKBR3 and to a minor extent in M21. Apoptosis was not detected when macrophages were treated with the different PPRHs. However, co-cultures of macrophages with the four cancer cell lines treated with PPRHs showed an increase in apoptosis. The order of fold-increase in apoptosis was HeLa > PC3 > SKBR3 > M21. This study demonstrates that PPRHs could be powerful pharmacological agents to use in immunotherapy approaches for the inhibition of PD-1 and PD-L1.

## Introduction

It is well known that the immune system can prevent the formation and progression of tumors by (i) eliminating viral infections that could lead to tumor formation, (ii) solving inflammation processes to avoid tumorigenesis and (iii) identifying and eliminating tumor cells depending on the expression of tumor-specific antigens (immune surveillance). Macrophages are one of the most important components involved in tumor elimination within the immune surveillance process [1,2]. Although macrophage cytotoxicity in tumors can be achieved by cytokine secretion, phagocytosis is the main process involved in tumor clearance [3,4]. One of the mechanisms of macrophages to trigger phagocytosis against tumor cells but avoiding normal tissues relies on distinguishing between non-self-molecules from self-molecules. When macrophages do not recognize tumor cells, these are not eliminated by the immune system, which represents one of the hallmarks of cancer [5].

During the last decade, immunotherapy approaches arose as innovative strategies to eradicate tumor cells. There is a wide spectrum of available immunotherapies ranging from cytokines such as IL-2 and IFN-α [6], cell-based therapies like vaccines [7] or adoptive cellular therapy to stimulate host’s immune system [8–11], and immune checkpoint blockade strategies using anti-CTLA-4 [12] or anti-PD-1 and anti-PD-L1 antibodies to trigger new immune responses against the tumor. Among them, PD-1/PD-L1 pathway has been the focus of extensive research in the recent years and it is considered one of the most successful advances in the history of cancer immunotherapy [12–14].

Programmed cell death protein 1 (PD-1) is an immunoinhibitory receptor that belongs to the CD28 family and it is expressed on B cells, activated T cells, dendritic cells, natural killer cells, tumor-infiltrating lymphocytes and activated monocytes. PD-1 has two main ligands: programmed cell death-ligand 1 (PD-L1) and programmed cell death-ligand 2 (PD-L2) [15,16]. However, research is more focused on PD-L1 because of its overexpression in different types of tumors [17,18].

In the tumor microenvironment, it is well established that PD-1 and its ligand PD-L1 are important in tumor progression and survival by escaping tumor neutralizing immune surveillance. Gordon *et al*. demonstrated that, by blocking PD-1/PD-L1 interaction with antibodies, the phagocytic potency of macrophages increased *in vivo*, thus reducing tumor growth in a cancer mouse model [19]. Giving the importance of PD-1/PD-L1 interaction to avoid phagocytosis, we used Polypurine reverse Hoogsteen hairpins (PPRHs) to silence both genes with the aim to provoke the elimination of tumor cells by macrophages.

PPRHs are non-modified single-stranded deoxyoligonucleotides formed by two antiparallel polypurine stretches linked by a pentathymidine loop. The intramolecular linkage consists of reverse Hoogsteen bonds that are formed between guanines and adenines, originating the hairpin structure. PPRHs can bind to polypyrimidine domains in the double-stranded DNA (dsDNA) via Watson-Crick bonds, thus displacing the fourth strand of the dsDNA and producing a triplex structure. That conformation leads to a transcriptional disruption that provokes the gene silencing effect [20,21]. Therefore, it is essential for PPRH design to find polypyrimidine tracts within the target gene sequence, which are mainly present in promoter or intronic regions [22].

In a previous study, we used this technology to conduct an immunotherapy approach based on silencing the *SIRPα* gene in macrophages and the *CD47* gene in breast cancer MCF-7 cells, to avoid their interaction and provoke the elimination of tumor cells by macrophages in co-culture experiments [23]. In addition, we demonstrated that PPRHs can act as pharmacological agents without causing hepatotoxicity or nephrotoxicity [24].

The aim of the present study was to eliminate tumor cells by macrophages in co-culture experiments by decreasing both the levels of PD-1 in macrophages and those of PD-L1 in different cancer cells using PPRHs and to evaluate the involvement of apoptosis in this approach.

## Materials and methods

### Cell culture and PMA induced differentiation

Prostate cancer PC3, melanoma M21, ovarian cancer HeLa, breast cancer SKBR3, and monocyte THP-1 cell lines were grown in Ham´s F-12 medium supplemented with 10% fetal bovine serum (both from Gibco, Barcelona, Spain) at 37°C in a 5% CO_2_-controlled humidified atmosphere. Trypsinization of cancer cells was performed using 0.05% Trypsin in PBS 1X (154 mM NaCl, 3.88 mM H_2_NaPO_4_ and 6.1 mM HNaPO_4_ pH 7.4) (Sigma-Aldrich, Madrid, Spain). THP-1 monocytes grew on suspension.

THP-1 cells were incubated with 2 ng/mL phorbol12-myristate 13-acetate (PMA) (Sigma-Aldrich, Madrid, Spain) for differentiation into macrophages. This concentration was chosen due to the patterns of pro-inflammatory cytokines and surface marker levels observed after three days of differentiation [23]. We routinely checked THP-1 differentiation by monitoring their adhesion to the plate and changes in cell morphology.

### Design of PPRHs

PPRHs were designed using The Triplex Oligonucleotide Target Sequence Search Software (http://utw10685.utweb.utexas.edu/tfo/, Austin, Texas, USA).

PPRHs were synthesized as non-modified desalted oligodeoxynucleotides by Sigma-Aldrich (HaverHill, United Kingdom). Lyophilized PPRHs were resuspended in sterile Tris-EDTA buffer (1 mM EDTA and 10 mM Tris, pH 8.0) (Sigma-Aldrich, Madrid, Spain) and stored at −20°C until use.

As a negative control, we used a Watson-Crick hairpin (Hp-WC) that forms intramolecular Watson–Crick bonds instead of reverse Hoogsteen bonds, and therefore the polypurine domain of the hairpin cannot bind to the polypyrimidine target sequence in the DNA.

**Fig 1:**
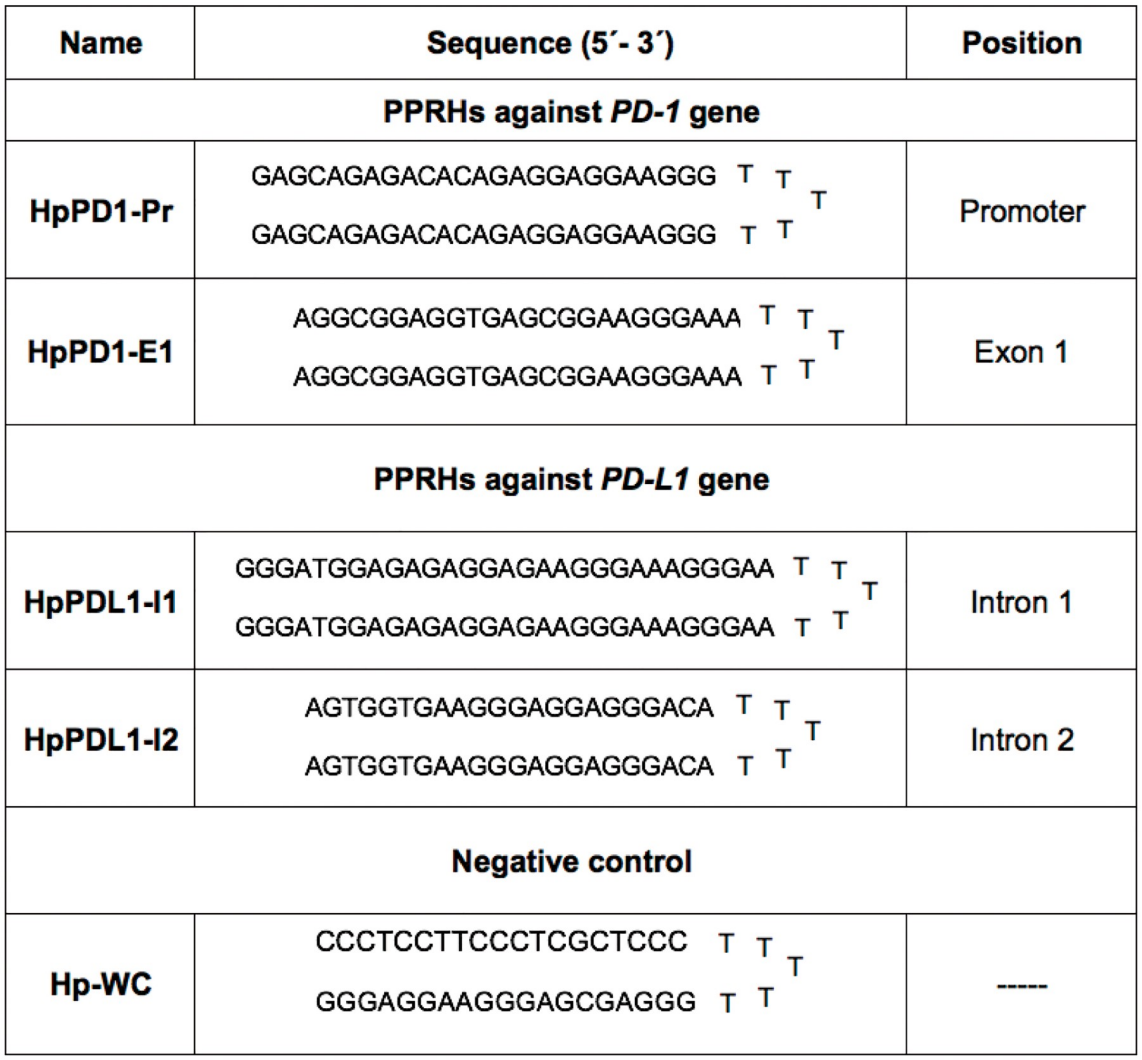
PPRHs designed against *PD-1* and *PD-L1* genes, as well as the negative control hairpin. Abbreviations are (i) Hp, hairpin; (ii) I, intron; (iii) Pr, promoter; (iv) E, exon. WC stands for the Watson-Crick negative control.

The sequences of the PPRHs and the negative control hairpin and their abbreviations are described in Fig 1.

### Transfection of PPRHs

Cells were plated in 6-well dishes. Transfection consisted in mixing 100 nM of PPRH with 10 μM of the cationic liposome N-[1-(2,3-dioleoyloxy)propil]-N,N,N-trimethylammonium methylsufate (DOTAP) (Biontex, München, Germany) in a final volume of 200 μL of culture medium. The mixture was incubated for 20 min at room temperature. Finally, the PPRH/liposome complex was added to the cells to attain a final volume of 1 mL.

### RNA extraction

Total RNA was extracted from PC3 and THP-1 cells 24 h and 48 h after transfection, respectively, using TRIzol (Life Technologies, Barcelona, Spain) following the manufacturer’s specifications. RNA was quantified by measuring its absorbance at 260 nm using a NanoDrop ND-1000 spectrophotometer (Thermo Fisher Scientific, Barcelona, Spain).

### Reverse transcription

cDNA was synthesized by reverse transcription in a 20 μl reaction mixture containing 1 μg of total RNA, 125 ng of random hexamers (Roche, Madrid, Spain), 500 μM of each dNTP (Panreac Applichem, Barcelona, Spain), 2 μL of 10X buffer, 20 units of RNAse inhibitor and 200 units of Moloney murine leukemia virus reverse transcriptase (Last three from Lucigen, Wisconsin, USA). The reaction was incubated at 42°C for 1 h.

### Real-time PCR

The StepOnePlus Real-Time PCR Systems (Applied Biosystems, Barcelona, Spain) was used to perform these experiments. The primer sequences to determine *PD1* mRNA levels were 5’GGATTTCCAGTGGCGAGAGA3’ and 5’CAGACGGAGTATGCCACCATT3’. TATA box binding protein (TBP) was used as endogenous control and the primer sequences were 5’GAGCTGTGATGTGAAGTTTCC3’ and 5’TCTGGGTTTGATCATTCTGTAG3’. The reaction was performed in a final volume of 20 μl, containing 1 X SYBR Universal PCR Master mix (Applied Biosystems, Barcelona, Spain), 0.25 μM of reverse and forward primers (Sigma-Aldrich, Madrid, Spain), 5 μl of cDNA and H_2_O mQ. PCR cycling conditions were 10 min denaturation at 95°C, 40 cycles of 15 s at 95°C and 1 min at 64°C.

To determine *PD-L1* mRNA levels in PC3 cells, *PD-L1* Taqman probe (Assay ID: Hs00204257_m1) was used. Cyclophilin A Taqman probe (PPIA) (Assay ID: Hs04194521-s1) was used as endogenous control. The reaction contained 1x TaqMan Universal PCR Master mix, 1x TaqMan probe (both from Applied Biosystems, Barcelona, Spain), 3 μL of cDNA and H_2_O mQ to a final volume of 20 μL. PCR cycling conditions were 10 min denaturation at 95°C, followed by 40 cycles of 15 s at 95°C and 1 min at 60°C.

The mRNA quantification was performed using the ΔΔCt method, where Ct is the threshold cycle that corresponds to the cycle where the amount of amplified mRNA reaches the threshold of fluorescence. Data were expressed as mRNA levels relative to the cells treated with the negative control Hp-WC.

### Western blot analyses

Total protein extracts from PC3 cells (90,000) were obtained 24 h after transfection. Cells were washed once with PBS 1X and collected by scrapping in 100 μL of Lysis buffer [150 mM NaCl, 1 mM EDTA, 50 mM Tris-HCl pH 8.0, 1.0% Igepal CA-630 (NP-40), 0.5% sodium deoxycholate, 0.1% sodium docecyl sulfate, 1 mM PMSF, 10 mM NAF and Protease inhibitor cocktail (all from Sigma Aldrich, Madrid, Spain)]. Cell debris was removed by centrifugation (12,000 x g at 4°C for 10 min).

In the case of THP-1 monocytes (90,000), cells were collected 48 h after transfection and centrifuged for 5 min at 800 x g at room temperature. Then, cells were resuspended in 50 μL of RIPA buffer [150 mM NaCl, 5 mM EDTA, 50 mM Tris-HCl pH 7,4, 1.0% Igepal CA-630 (NP-40), 100 μg/mL PMSF and Protease inhibitor cocktail (all from Sigma Aldrich, Madrid, Spain)]. Cell lysate was kept on ice for 30 min, vortexing every 10 min. Cell debris was removed by centrifugation at 12,000 x g at 4°C for 10 min. The Bradford method was used to determine protein concentration using bovine serum albumin as a standard.

Whole cell extracts were resolved in 10% SDS-polyacrylamide gels and transferred to PVDF membranes. The blocking solution was 5% Blotto. Membranes were probed during 90 min at room temperature with primary antibodies against PD-L1 (1:250 dilution; PA5-28115, Thermo Fisher Scientific, Barcelona, Spain), PD-1 (Pdcd-1L1, 1C10; 1:50 dilution; sc-293425, Santa Cruz Biotechnology, Heidelberg, Germany) and GAPDH (1:200 dilution; sc-47724, Santa Cruz Biotechnology, Heidelberg, Germany) to normalize the results. Detection was achieved by secondary HRP-conjugated antibodies: anti-rabbit (1:1000 dilution; Dako, Denkmark) for PD-L1 and anti-mouse (1:1000; sc-516102, Santa Cruz Biotechnology, Heidelberg, Germany) for PD-1 and GAPDH. Chemiluminescence was detected with Image Quant LAS 4000 mini technology (GE Healthcare, Barcelona, Spain). Quantification was performed using Image Studio Lite Software. Data were represented as protein levels relative to the control cells (untransfected cells).

### Cell titration

Before performing the co-culture experiments, we set up the number of cells to be used for each cell line. Cell titration was carried out by increasing either the number of macrophages or the number of cancer cells. In the case of macrophages/PC3 co-cultures, we opted to start with 80,000 PC3 cells that were cultured with an increasing number of macrophages, observing that the best ratio was obtained with 10,000 cells (Table 1). Regarding macrophages/SKBR3 co-cultures, we selected 10,000 macrophages with an increasing number of SKBR3 cells and the best ratio was established at 1:6 (Table 2). When using macrophages/M21 co-cultures, since M21 were more resistant to the treatment, we opted for increasing the number of macrophages to 60,000, thus establishing a macrophages/M21 ratio of 1:1. Finally, in the macrophages/HeLa co-cultures, the experiments were performed using the same conditions that with macrophages/PC3 co-cultures.

**Table 1:** Co-culture titration of PC3 cells with increasing number of macrophages.

Macrophages/PC3 Co-culture titration with 80,000 PC3 cells	
N° of macrophages	Cell viability (%)
1,000	66%
5,000	32%
10,000	10%
20,000	60%

**Table 2:** Co-culture titration of macrophages with increasing number of SKBR3 cells.

Macrophages/SKBR3 Co-culture titration with 10,000 macrophages	
N° of SKBR3 cells	Cell viability (%)
60,000	12%
90,000	22%
120,000	32%

### Co-culture experiments

**Fig 2:**
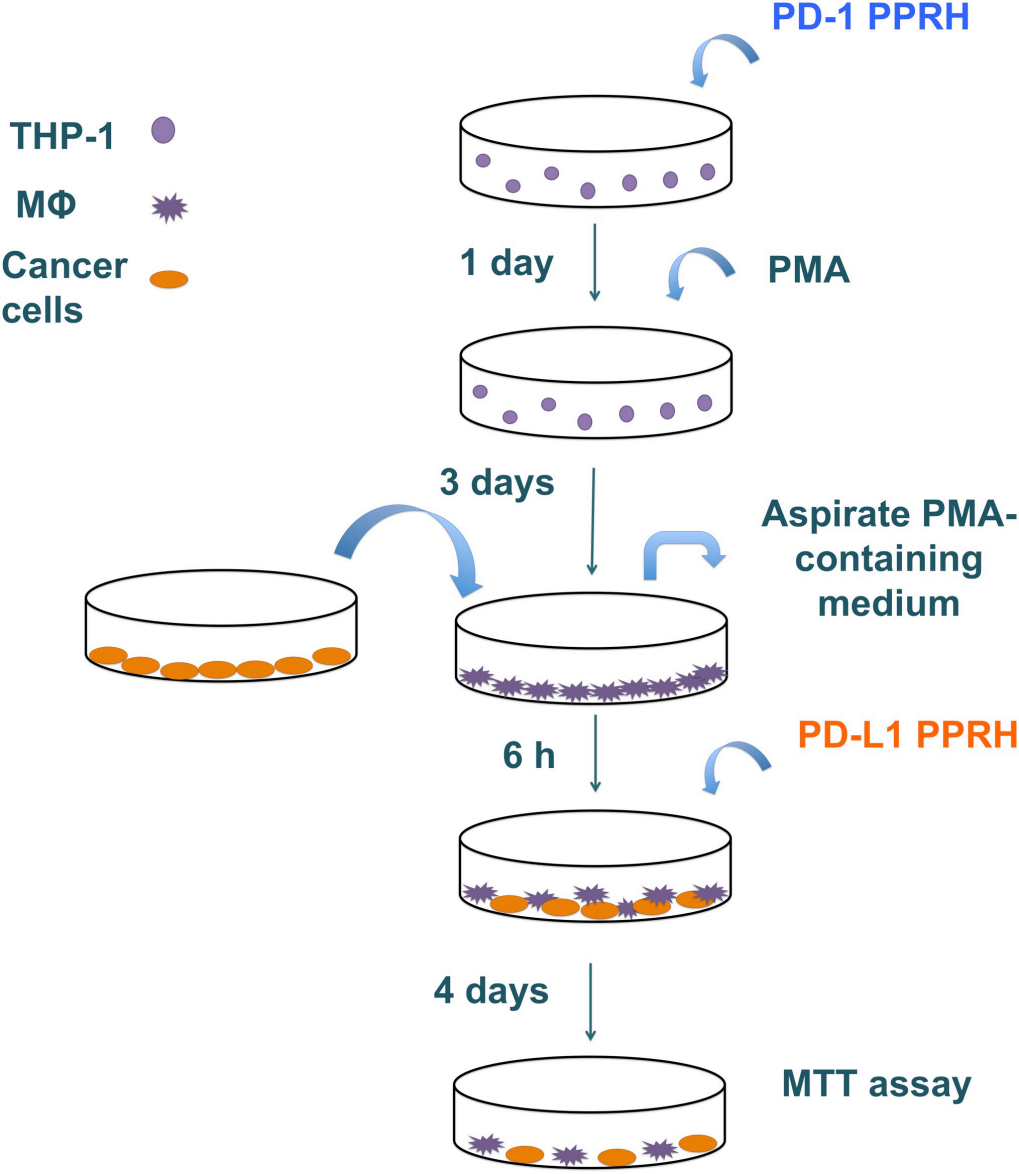
Schematic representation of co-culture experiments. THP-1 cells were plated and immediately transfected with PPRHs against *PD-1*. After 24 h, transfected THP-1 cells were differentiated to macrophages (Mϕ) with PMA for 3 days. At that time, the PMA-containing medium was replaced with fresh medium and cancer cells were added to the dishes containing the macrophages. After 6 h, cells were transfected with different PPRHs against *PD-L1*. Cell viability (MTT) assay was performed 4 days after transfection with PPRHs against *PD-L1*.

THP-1 cells were plated in 6-well dishes and transfected with different PPRHs against *PD-1*. After 24 h, transfected THP-1 cells were treated with 2 ng/mL of PMA for differentiation. Three days after PMA treatment, the PMA-containing medium was replaced with fresh medium and different number of cancer cells were added to the plates containing the macrophages, as indicated in Table 3. After 6 h, cells were transfected with different PPRHs against *PD-L1*. Cell viability was assessed 4 days after the last transfection by MTT assays. The co-culture procedure is depicted in Fig 2.

**Table 3:** Number of macrophages and cancer cells plated in each co-culture experiment.

Cancer cell line	Number of cancer cells in co-culture experiments	Number of macrophages in co-culture experiments
PC3	80,000	10,000
M21	60,000	60,000
SKBR3	60,000	10,000
HeLa	80,000	10,000

### MTT assays

Four days after the last transfection, 500 μg/mL of 3-(4,5-Dimethylthiazol-2-yl)-2,5-Diphenyltetrazolium Bromide (MTT) and 100 μM of sodium succinate (both from Sigma-Aldrich, Madrid, Spain) were added to the culture medium and incubated for 2.5 h at 37°C for the reaction. After incubation, the medium was removed and the solubilization reagent (0.57% acetic acid and 10% sodium dodecyl sulfate in DMSO) (Sigma-Aldrich, Madrid, Spain) was added. Cell survival was measured at 570 nm in a Modulus Microplate luminometer (Turner BioSystems; Promega, Madrid, Spain). Results were expressed as the percentage of cell survival relative to cells transfected with the negative control (Hp-WC).

### Apoptosis assay

Apoptosis was determined by the rhodamine method: 48 h after the last transfection, rhodamine 123 (final concentration 5 μg/mL) (Sigma-Aldrich, Madrid, Spain) was added for 30 min. Then, cells were collected, centrifuged at 800 x g at 4°C for 5 min and washed once in PBS. The pellet was resuspended in 500 μL of cold PBS and Propidium Iodide at a final concentration of 5 μg/mL (Sigma-Aldrich, Madrid, Spain) was added. Flow cytometry analyses were performed in a Beckman Coulter CyAn ADP cytometer (Beckman Coulter Inc., Madrid, Spain) and data were analyzed using the software Summit v4.3. The percentage of propidium iodide-negative and rhodamine-negative cells corresponded to the apoptotic population. Data were expressed as the apoptosis fold-increase levels relative to the co-culture transfected with the negative control Hp-WC.

### Statistical analyses

Statistical analyses were carried out using GraphPad Prism 5 (GraphPad Software, California, USA). All data are shown as the mean ± SEM of three independent experiments. Statistical significance was determined using one-way analysis of variance, followed by Dunnett’s multiple comparison test. In the apoptosis experiments, a two-way ANOVA with Sidak’s multiple comparisons test was used. Differences were considered significant when p < 0.05.

## Results

### Effect of PPRHs on *PD-1* and *PD-L1* mRNA and protein levels

**Fig 3:**
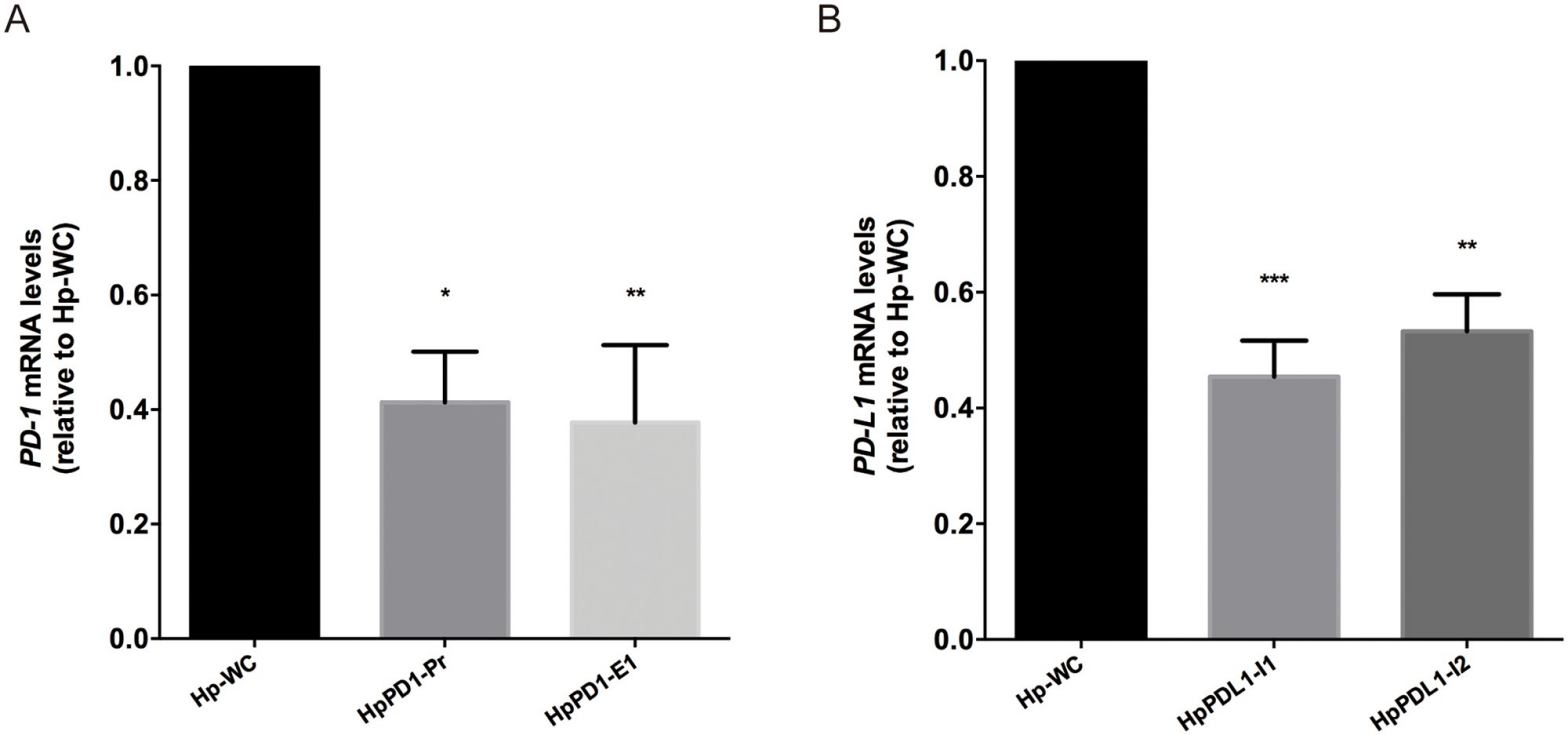
Effect of PPRHs on *PD-1* and *PD-L1* mRNA levels. A) THP-1 cells (90,000) were transfected with two PPRHs against *PD-1* and mRNA levels were determined 48 h after transfection. B) PC3 cells (90,000) were transfected with two PPRHs against *PD-L1* and mRNA levels were assessed 24 h after transfection. mRNA levels are plotted relative to the cells treated with the negative control (Hp-WC). Data represent the mean ± SEM of three experiments. (*p < 0.05, **p < 0.01, ***p < 0.005).

**Fig 4:**
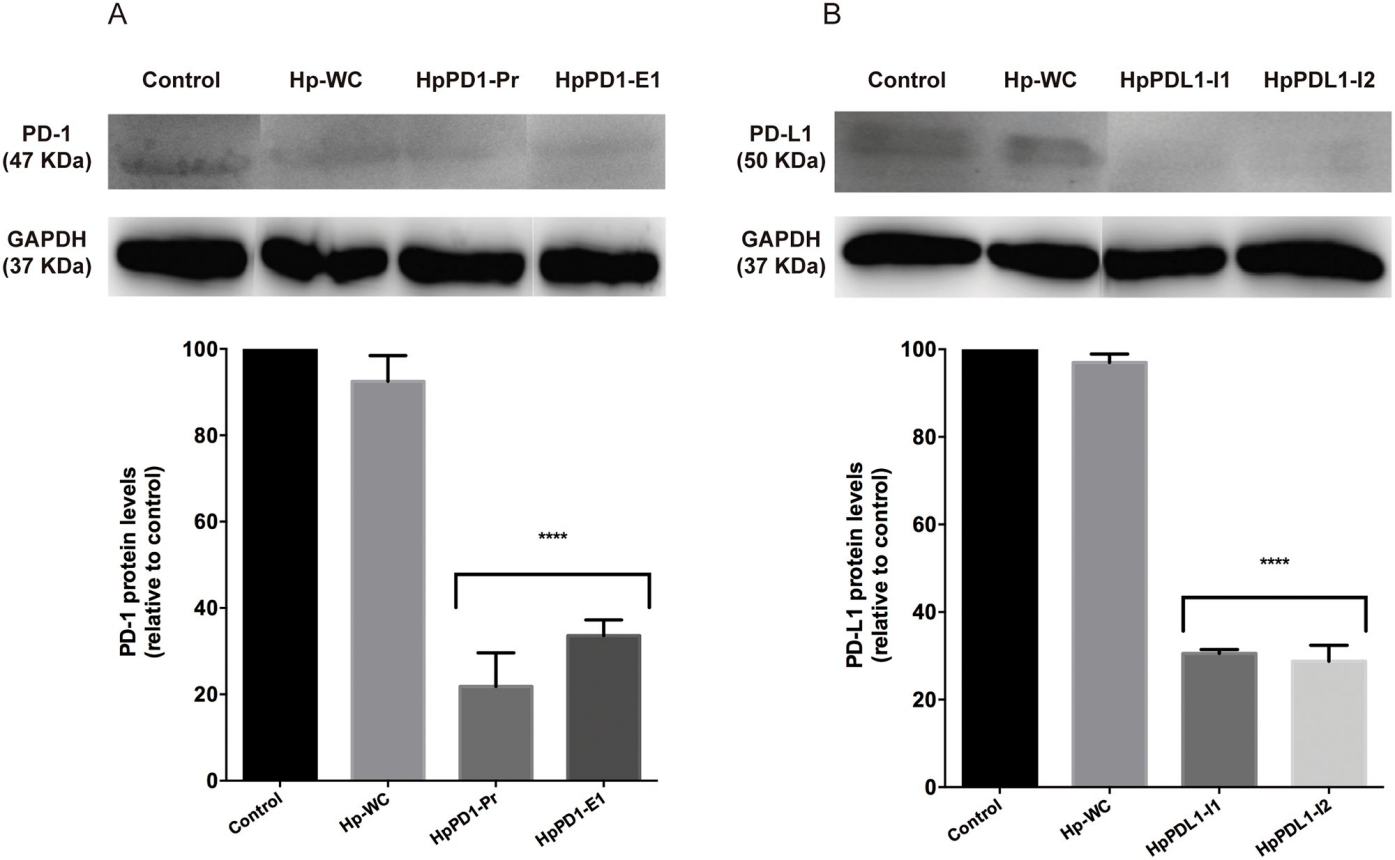
Effect of PPRHs on PD-1 and PD-L1 protein levels. A) THP-1 cells (90,000) were transfected with two PPRHs against PD-1 and protein extracts were obtained after 48 h. B) PC3 cells (90,000) were transfected with two PPRHs against PD-L1 and proteins were extracted after 24 h. Representative Western blot images of PD-1 and PD-L1 are shown. The quantification of the changes in protein levels were determined upon normalization with the signal corresponding to GAPDH protein. Non-transfected cells and cells treated with the negative control hairpin were used as controls. Data represent the mean ± SEM of three experiments. (****p < 0.001).

PPRHs against *PD-1* were tested in THP-1 cells. *PD-1* mRNA levels were determined upon cell incubation with two different PPRHs against *PD-1*, HpPD1-Pr and HpPD1-E1, whose sequences are shown in Fig 1. These PPRHs were able to decrease *PD-1* mRNA levels by 2.4 and 2.7-fold, respectively, compared to the negative control Hp-WC (Fig 3A). On the other hand, when targeting *PD-L1* in PC3 cells, two different PPRHs were used, HpPDL1-I1 and HpPDL1-I2 (Fig 1), which decreased *PD-L1* mRNA levels by 2.2 and 1.8-fold, respectively, compared to the negative control (Fig 3B). When analyzing PD-1 protein levels in THP-1 cells, HpPD1-Pr and HpPD1-E1 decreased PD-1 protein by 78% and 66%, respectively, relative to the control (Fig 4A). HpPDL1-I1 and HpPDL1-I2 decreased PD-L1 protein levels in PC3 cells by 69% and 71%, respectively, compared to the control (Fig 4B).

### Effect of PPRHs on cell viability in PC3 and THP-1 cells

**Fig 5:**
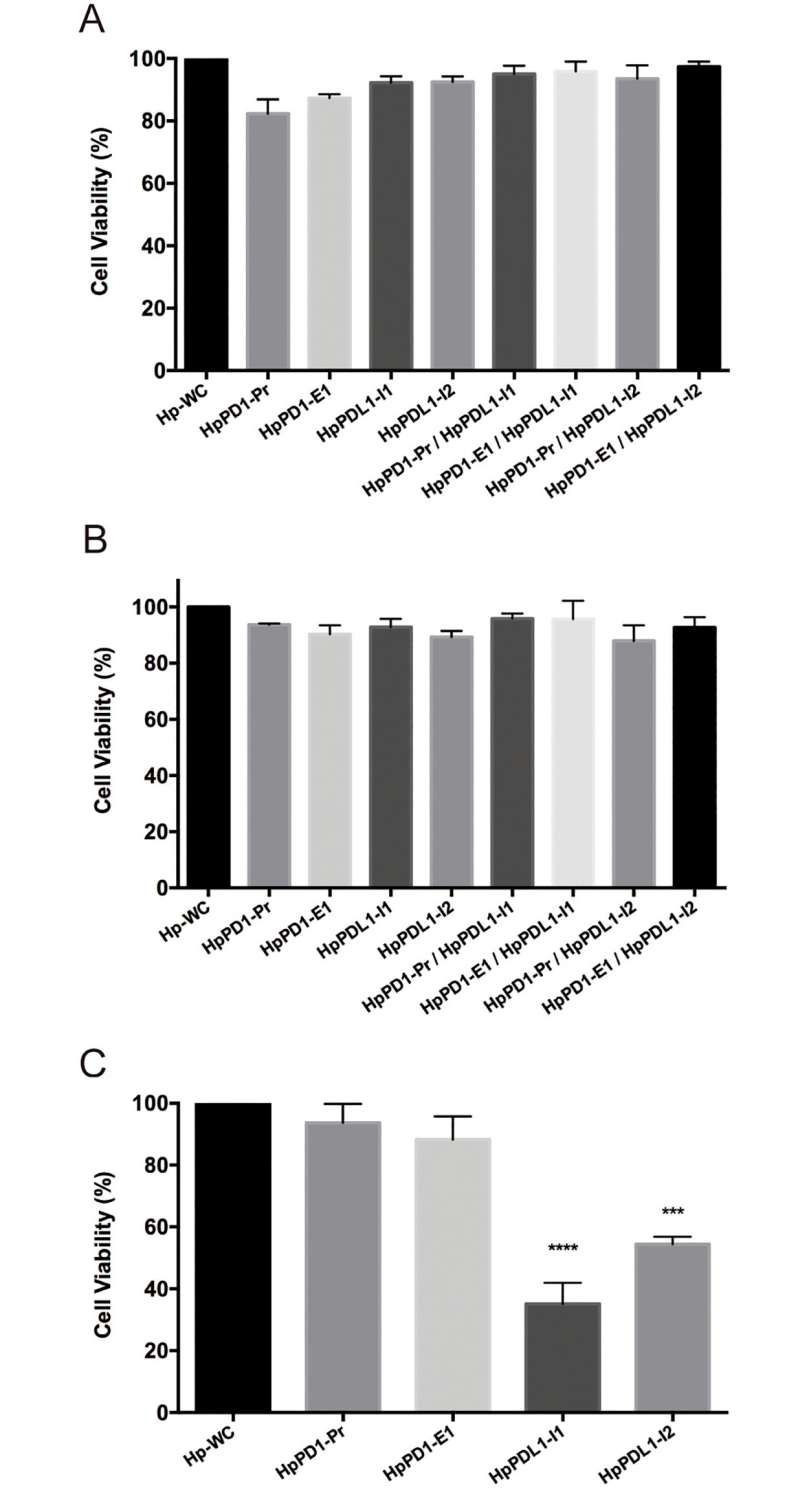
Effect on cell viability upon incubation with PPRHs against *PD-1* and *PD-L1*. A) THP-1 cells (10,000) were treated with PPRHs against *PD-1*, *PD-L1* or in combination. B) Macrophages (10,000) were treated with PPRHs against *PD-1*, *PD-L1* or in combination. C) PC3 cells (80,000) were transfected with PPRHs against *PD-1* and *PD-L1*. Cell viability was assessed 5 days after transfection. Cells treated with the negative control hairpin (Hp-WC) were used as control. Data represent the mean ± SEM of three independent experiments performed on different days. (***p<0.005, ****p< 0.001).

We determined whether the different PPRHs against *PD-1* and *PD-L1* could provoke, on their own, any cytotoxic effect in THP-1 and PC3 cells. The transfection of the different PPRHs separately and their combinations did not cause any significant effect on cell viability in either THP-1 (Fig 5A) or macrophages (Fig 5B). However, in PC3 cells, HpPDL1-I1 and HpPDL1-I2 directed against *PD-L1*, provoked a decrease in cell viability of 65% and 45%, respectively, confirming the role of PD-L1 in tumor cell progression (Fig 5C). In contrast, PPRHs against PD-1 did not cause any effect in PC3 cells (Fig 5C).

### Effect of PPRHs on PC3 cell viability in co-culture experiments

**Fig 6:**
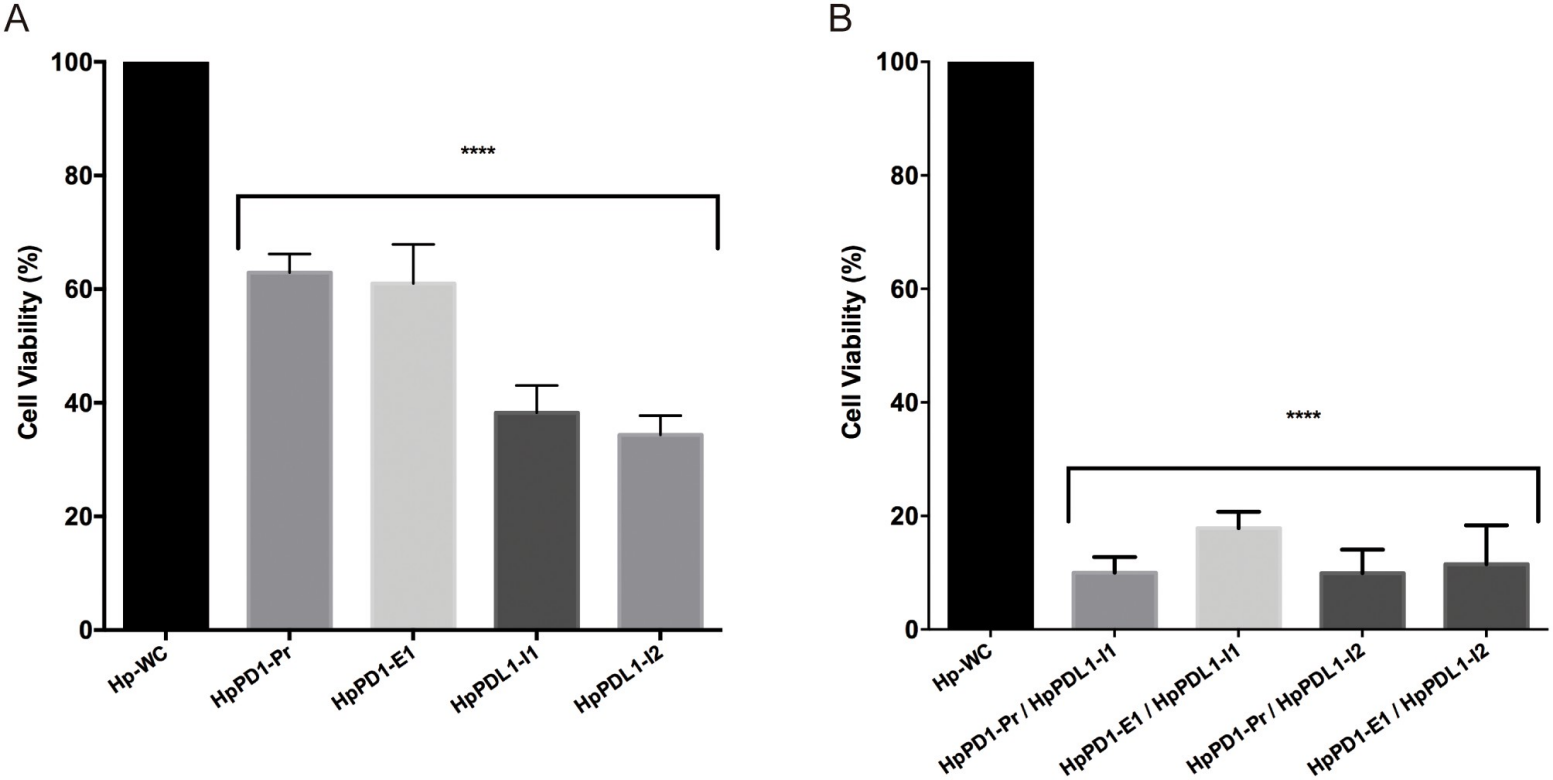
Macrophages/PC3 co-culture experiments. A) Effect on cell viability of PPRHs against *PD-1* transfected only in 10,000 macrophages or PPRHs against *PD-L1* transfected only in 80,000 PC3 cells, in co-culture experiments. B) Effect on cell viability, in co-culture experiments, of PPRHs against PD-1 transfected in macrophages plus PPRHs against PD-L1 transfected in PC3 cells, making a total of four combinations of PPRHs. Co-cultures incubated with the negative control hairpin (Hp-WC) were used as controls. Data represent the mean ± SEM of three independent experiments. (****p< 0.001).

First, we tested the effect of HpPD1-Pr and HpPD1-E1 against *PD-1* and HpPDL1-I1 and HpPDL1-I2 against *PD-L1* in co-culture experiments with macrophages and PC3 cells. When silencing *PD-1* in macrophages with HpPD1-Pr or HpPD1-E1, macrophages were able to kill 37% and 39% of PC3 cells, respectively (Fig 6A). When *PD-L1* was silenced with HpPDL1-I1 or HpPDL1-I2 in PC3 cells, 62% and 66% of the cells were killed by macrophages (Fig 6A). To assess whether the inhibition of both target genes could lead to an enhanced effect, macrophages and PC3 cells were transfected together in co-culture experiments with the four combinations of PPRHs such as each of the two hairpins against *PD-1* was combined with each one against *PD-L1*. In these conditions, we observed that the best combination of PPRHs against *PD-1*/*PD-L1* was HpPD1-Pr/HpPDL1-I1 that provoked 90% cell death in co-culture (Fig 6B).

### Effect of PPRHs on cell viability in co-culture experiments with M21, HeLa and SKBR3 cells

**Fig 7:**
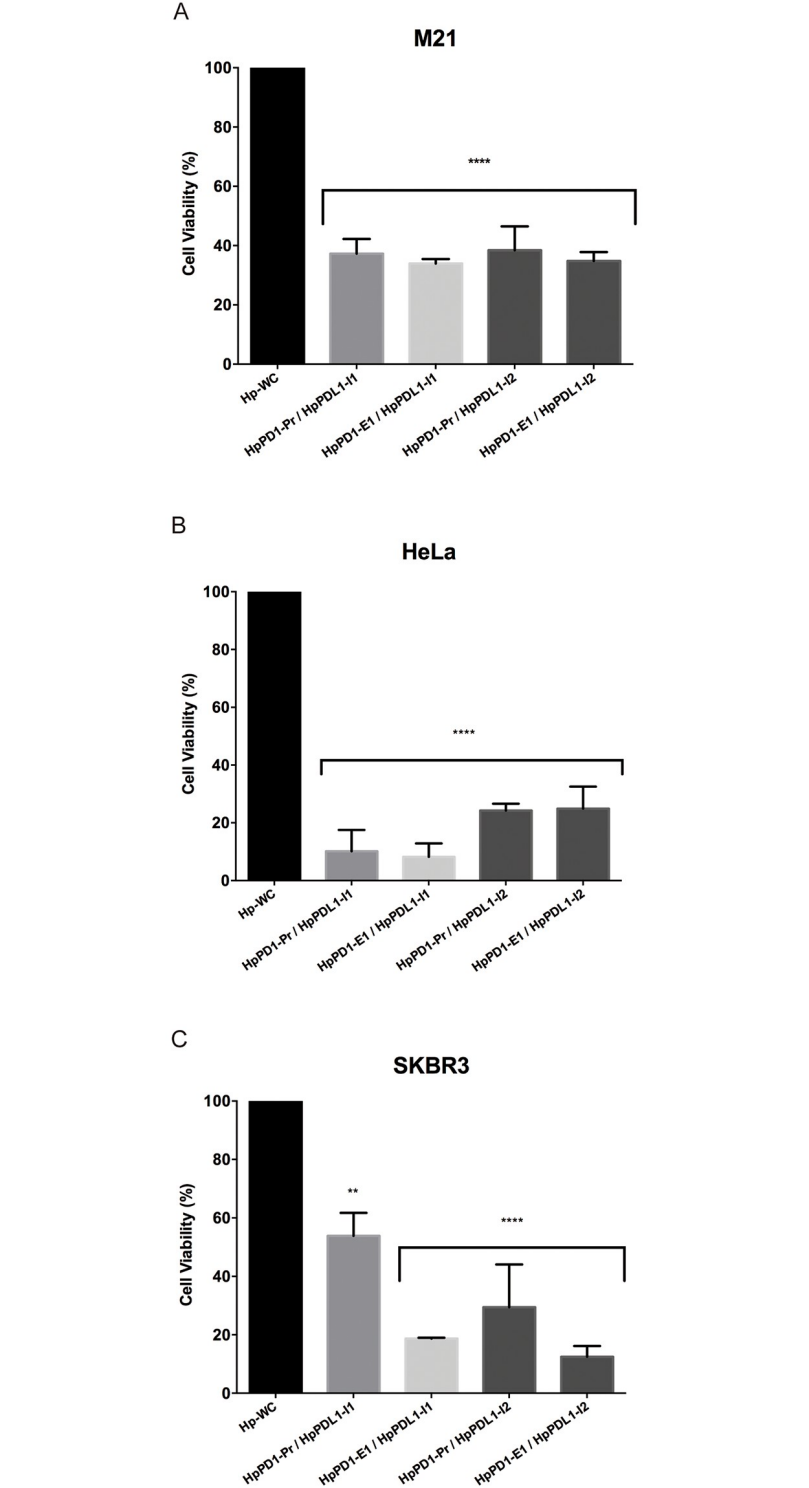
Co-culture experiments with other cancer cell lines. M21 (60,000) (A), HeLa (80,000) (B) or SKBR3 (60,000) (C) cells were co-cultured with 60,000, 10,000 and 10,000 macrophages, respectively. Macrophages were treated with either HpPD1-Pr or HpPD1-E1 whereas cancer cells were transfected with either HpPDL1-I1 or HpPDL1-I2 against *PD-L1*. Co-cultures treated with the negative control hairpin (Hp-WC) were used as control. Data represent the mean ± SEM of at least three experiments. (**p < 0.01, ****p < 0.001).

To extend the results observed in PC3 cells, we incubated all the combinations of PPRHs against *PD-1* and *PD-L1* in three other cancer cell lines, M21, HeLa and SKBR3, in co-culture experiments. In M21 cells, the best combination of PPRHs was HpPD1-E1/HpPDL1-I1, which provoked 65% cell death (Fig 7A). In HeLa cells, the HpPD1-E1/HpPDL1-I1 combination led to a reduction of 92% in cell viability (Fig 7B). Finally, the combination HpPD1-E1/HpPDL-I2 in SKBR3 cells was able to reduce cell viability by 88% (Fig 7C).

### Level of apoptosis in co-culture experiments with PC3, M21, HeLa and SKBR3 cells

**Fig 8:**
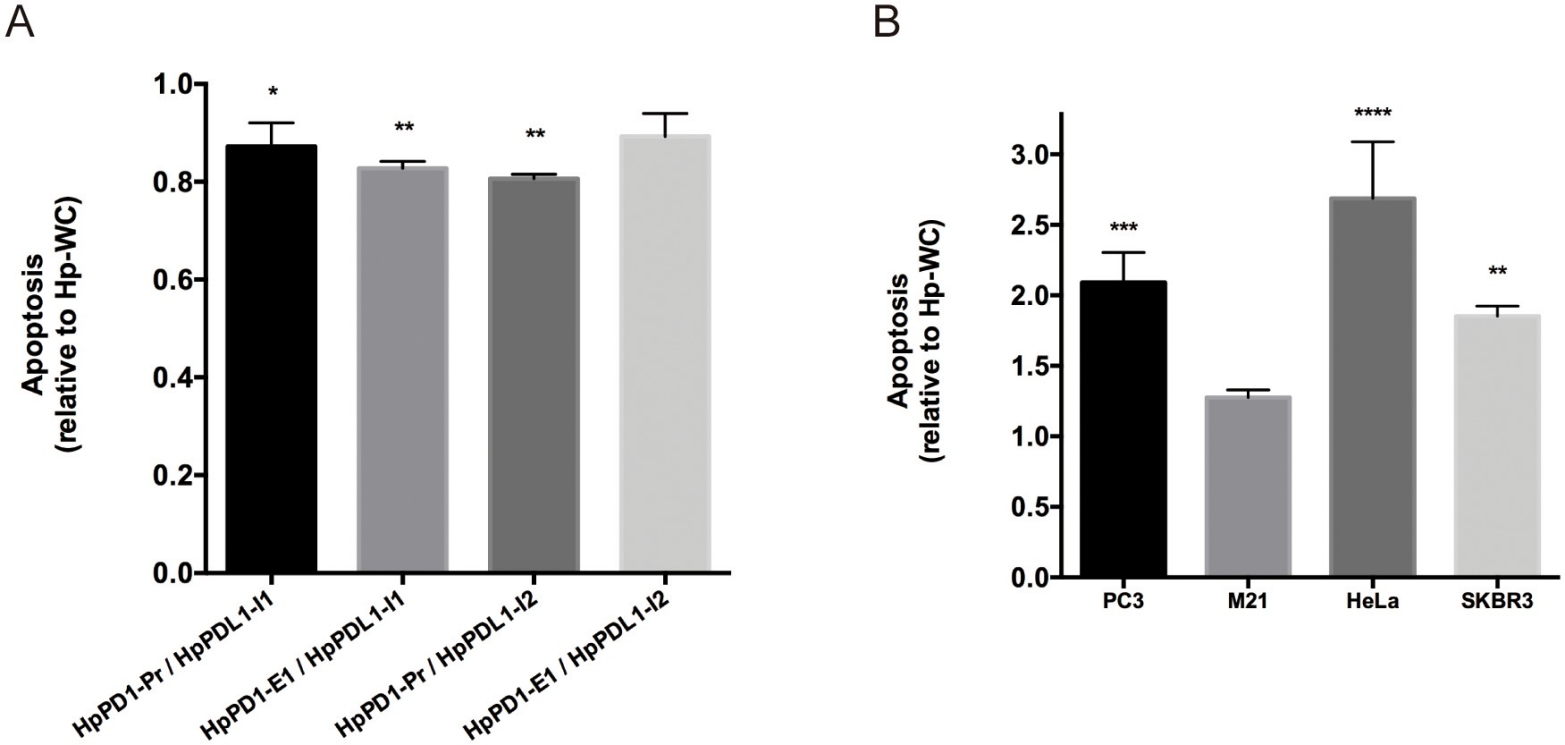
Apoptosis determination. A) Apoptotic levels in macrophages treated with the different combinations of PPRHs. B) Apoptotic levels in co-culture experiments using the most effective combination of PPRHs for each cancer cell line. Changes in apoptosis are represented relative to co-cultures transfected with the negative control hairpin (Hp-WC). Data represent the mean ± SEM of three experiments performed on different days. (*p < 0.05, **p < 0.01, ***p < 0.005, ****p < 0.001).

To gain insight into the mechanism that provoked cancer cells death in the co-culture experiments, we determined the levels of apoptosis in the different cell lines upon 48 hours of incubation with the different PPRHs. The most effective combinations of PPRHs against *PD-1* and *PD-L1* determined in the cell viability assays for each cancer cell line were selected for the apoptosis studies. First of all, we checked the effect on apoptosis of the different combinations of PPRHs in macrophages. We did not observe any increase in the apoptotic levels (Fig 8A), thus demonstrating that macrophages were not affected by the transfection of the different PPRHs. However, co-cultures of macrophages with either PC3, HeLa or SKBR3 cells showed 2.1, 2.7 and 1.8-fold increase in apoptosis, respectively, when compared to their respective negative controls (Fig 8B). Finally, co-culture of macrophages with M21 cancer cell line showed a moderate increase (1.3-fold) in the apoptotic population after the treatment (Fig 8B), in accordance with the observed results in cell viability.

## Discussion

In this work we describe an immunotherapy approach using PPRHs directed against *PD-1* and *PD-L1* in PMA-differentiated THP-1 cells and PC3 cancer cells, respectively, to favor the elimination of tumor cells by macrophages. It has been previously demonstrated that these molecules represent a novel gene silencing tool against different cancer targets, both *in vitro* [20,25–27] and *in vivo* [28]. In addition, PPRHs are more stable, showing a half-life much longer than that of siRNAs, and they do not activate the innate inflammatory response [29]. We have used this gene silencing tool to conduct an immunotherapy approach against both *CD47* in MCF-7 cells and *SIRPα* in macrophages, respectively, achieving a large decrease in cell viability [23].

In the present study, we showed that different cancer cells were killed by macrophages in co-culture experiments upon silencing of *PD-1*/*PD-L1* interaction with four different PPRHs, whereas tumor cells remain unaffected when treated with the negative control.

A first step consisted in determining at the molecular level that the designed PPRHs were able to decrease the expression at the level of mRNA and protein of *PD-1* and *PD-L1* in THP-1 monocytes and PC3 cells, respectively, thus demonstrating the specific gene silencing effect of the four PPRHs used.

An important point was to verify whether any of the four PPRHs used in the study was able to produce a cytotoxic effect *per se* due to the specific silencing of either *PD-1* or *PD-L1* in both THP-1 and PC3 cells, in the absence of co-culture. The rationale for this control was to state the contribution of macrophages in the co-culture experiments to eliminate cancer cells after silencing PD-1 and PD-L1 in THP-1 and PC3 cells, respectively, and not by the effect that the PPRHs could provoke by themselves. In the case of THP-1 cells and macrophages, none of the four PPRHs, two against *PD-1* and two against *PD-L1*, produced a significant effect on cell viability. However, in PC3 cells, the two PPRHs directed against *PD-L1* were able to decrease cell viability by themselves. It has been reported that, aside from avoiding tumor immunity, PD-1/PD-L1 inhibition has also cancer cell-intrinsic functions that promote tumor growth and survival such as mTOR signaling. Therefore, suppressing either PD-1 or PD-L1 could attenuate the growth of PD-L1^+^ cancer cells [30,31]. In this direction, in a recent study Li *et al*. reported that the inhibition of *PD-L1* with a siRNA in gastric cancer cells suppressed cell proliferation, migration and tumorigenicity both *in vitro* and *in vivo* [32]. Another study also demonstrated that silencing *PD-L1* in colon cancer cells with a siRNA reduced cell progression and led to an increase in apoptosis [33]. Similarly, Song *et al*. reported that knocking down PD-L1 in pancreatic ductal adenocarcinoma decreased cell proliferation [34]. Finally, a recent study of Kwak and collaborators showed that silencing of PD-L1 with a siRNA in melanoma cells provoked a 25% decrease in cell viability *in vitro*. When the siRNA complexed with a polymeric carrier was injected in a xenograft mouse model, tumor growth was reduced by 60% approximately [35].

Cerignoli and collaborators showed that stimulated PBMCs previously treated with an anti-PD-1 antibody provoked a maximum cytolysis of 80% in PC3 cells later in co-culture experiments [36]. In this direction, in our co-culture approach, when *PD-1* was silenced in macrophages with non-transfected PC3 cells, the reduction in cell viability was about 40%.

When comparing the effect of silencing *PD-L1* with HpPDL1-I2 in PC3 cells alone with that produced in PC3 cells co-cultured with non-transfected macrophages, the reduction of cell viability in the latter was 20% higher. Since PD-1/PD-L1 interaction prevents the phagocytosis of tumor cells by macrophages, our hypothesis was to achieve an additional effect by inhibiting PD1/PD-L1 interaction acting on both genes in their respective cell lines, thus increasing the efficacy of the immunotherapeutic treatment. Therefore, an important conclusion of our work is that silencing *PD-1* and *PD-L1* in THP-1 and PC3 cells, respectively, led to death of the vast majority of PC3 cells (90%) by macrophages in each of the four possible combinations of PPRHs.

At this point, we wanted to expand our results in PC3 cells to additional cancer cell lines. In this regard the same approach was applied in M21, HeLa and SKBR3 cancer cell lines, observing that silencing of PD-1 and PD-L1 in co-culture experiments produced a high degree of cell mortality in all cases. HeLa cells were the most affected by the treatment, followed by SKBR3 and M21 cells. Based on data from The Human Protein Atlas (https://www.proteinatlas.org/), both cervix and prostate tissues present a higher expression of PD-L1 compared with breast and skin tissues. For that reason, we believe that the differences in the outcome of the treatment in the different cancer cell lines could be due to the differential expression of *PD-L1*. Iwamura *et al*. described that when suppressing *PD-L1* expression with a siRNA in a lung adenocarcinoma cell line, the specific lysis of tumor cells conducted by CD8+ T cells was 10% [37]. However, in our case, when silencing PD-L1 with PPRHs in PC3 cells, macrophages were able to kill two thirds (66%) of the cancer cell population. Juneja *et al*. also determined that *PD-L1* expression in murine colon adenocarcinoma MC38 cells inhibited CD8+ T cell response and cytotoxicity against tumor cells. However, CD8+ T cells were still able to kill tumor cells that did not express *PD-L1*, demonstrating its significant suppressing effect [38].

Another conclusion from our study is that apoptosis is part of the mechanism of killing in co-culture incubations with differentiated macrophages and cancer cells treated with their respective PPRHs targeting either PD-1 or PD-L1, in agreement with those reported in [36]. The levels of apoptosis in each cell line correlate with its observed decrease in cell viability.

There are currently two anti-PD-1 and three anti-PD-L1 antibodies approved for the treatment of different types of cancer and some other molecules are still in clinical trials [39]. Several anti-CD47 antibodies are also in Phase I clinical trials [40]. In a recent study, anti-CD47 and anti-PD-L1 monotherapies were used against tumor mice models. Although both treatments were able to reduce tumor size, combination of anti-CD47 and anti-PD-L1 treatments showed the greatest reduction, thus increasing the survival of the animals more than either monotherapy [19]. Therefore, CD47 and PD-L1 are good targets for immunotherapy strategies, as we also demonstrated using PPRHs against these genes in the present work and in [23].

In conclusion, we performed an *in vitro* immunotherapy approach based in silencing, by means of PPRHs, *PD-1* in macrophages and *PD-L1* in different cancer cells in co-culture experiments to inhibit their interaction, thus increasing the phagocytic potency of macrophages against the tumor. Therefore, this work extends the usage of PPRHs as alternative pharmacological agents in immunotherapy against PD-1 and PD-L1.
